# Trajectory Strategy Effects on the Material Characteristics in the WAAM Technique

**DOI:** 10.3390/mi14040827

**Published:** 2023-04-08

**Authors:** Tran Minh The Uyen, Pham Son Minh, Van-Thuc Nguyen, Thanh Trung Do, Vinh Tien Nguyen, Minh-Tai Le, Van Thanh Tien Nguyen

**Affiliations:** 1 HCMC University of Technology and Education, Ho Chi Minh City 71307, Vietnam; uyentmt@hcmute.edu.vn (T.M.T.U.);; 2 Department of Industrial Engineering and Management, National Kaohsiung University of Science and Technology, Kaohsiung 80778, Taiwan; 3 Falcuty of Mechanical Engineering, Industrial University of Ho Chi Minh City, Nguyen Van Bao Street, Ward 4, Go Vap District, Ho Chi Minh City 70000, Vietnam

**Keywords:** spiral trajectory, microstructure, tensile strength, elongation

## Abstract

The wire Arc Additive Manufacturing (WAAM) technique has evolved into a cutting-edge 3D printing technique. This study surveys the influences of trajectory on the characteristics of low-carbon steel samples generated by the WAAM technique. The results show that the grains in the WAAM samples are isotropic, with grain size numbers ranging from 7 to 12. Strategy 3, with a spiral trajectory, has the smallest grain size, while strategy 2, with a lean zigzag trajectory, has the largest. The variations in grain size are caused by differences in heat input and output during the printing process. The WAAM samples achieve a significantly higher UTS value than the original wire, demonstrating the WAAM technique’s benefit. Strategy 3, with a spiral trajectory, achieves the highest UTS value, 616.5 MPa, 24% higher than the original wire. The UTS values of strategy 1 (horizontal zigzag trajectory) and strategy 4 (curve zigzag trajectory) are comparable. WAAM samples have significantly higher elongation values than the original wire, with only 22% elongation. The sample with the highest elongation value, 47.2%, was produced by strategy 3. Strategy 2 has an elongation value of 37.9%. The value of elongation is proportional to the value of UTS. WAAM samples have average elastic modulus values of 95.8 GPa, 173.3 GPa, 92.2 GPa, and 83.9 GPa, corresponding to strategies 1, 2, 3, and 4. Only a strategy 2 sample has a similar elastic modulus value to the original wire. All samples have dimples on the fracture surface, indicating that the WAAM samples are ductile. These fracture surfaces’ equiaxial shape corresponds to the original microstructure’s equiaxial shape. The results provide the optimal trajectory for the WAAM products is the spiral trajectory, while the lean zigzag trajectory gains only modest characteristics.

## 1. Introduction

In recent decades, additive manufacturing (AM) has become popular [1,2,3]. In this technique, the products are usually added or fabricated layer by layer from 3D model data, which differs from the traditional subtractive methods. The technique’s most valuable feature is its ability to construct a complex object accurately. A 3D printer is easy to use if the 3D model data file is available, making it suitable for industrial and educational purposes. Moreover, 3D printing is also an advanced selection when manufacturers want to create rapid prototypes during the design process, as it can save time and cost. In fact, rapid prototyping is the initial application of 3D printing before it can be applied to mass production. Based on the way the raw materials are solidified, AM could be classified as fused deposition modeling (FDM), stereolithography (SL), selective laser sintering (SLS), electron beam melting (EBM), and wire arc additive manufacturing (WAAM). However, some drawbacks prevent it from becoming a more dominant technique, such as requiring much energy, time, and expensive equipment [4,5,6]. As a result, additive manufacturing applications are still limited in some areas, such as automotive, aerospace, biomedical, machinery, and robotics [7,8,9]. Wire shape materials save significantly more energy and time than powder travel materials. In accordance, it is more widely used in industries than powder materials.

WAAM technique uses an electrical arc as a heating source for melting wires [10,11,12,13]. WAAM could produce parts with large sizes, low costs, and high-efficiency thanks to the accessibility of wires, arc sources, and mechanical devices [14,15]. As a result, many authors have discussed the WAAM process’s parameter optimization [16,17,18,19,20]. Ke et al. [21], for instance, suggested that ultra-high-frequency pulses were used to refine the grain structure and produce a high-quality WAAM product. With the assistance of ultra-high-frequency pulsed, the molten pool of the NiTi shape memory alloys is vibrated, leading to the refinement of the grain structure. Henckell et al. [22] tried to minimize energy input by employing a short arc welding regime, thereby improving the shape and microstructure of WAAM samples. Applying a brief arc welding regime, the input energy might reduce from 10% to 40%. As a result, the cooling rate improves, leading to a decrease in grain size [23].

Bambach et al. [24] investigated the machining process of titanium alloy Ti-6Al-4 V using metal forming and the WAAM technique for aerospace products. Firstly, a semi-finished shape is created via the WAAM process. Then, this part is forged and heat-treated. The ultimate tensile strength and the ductility of the final product are as good as the traditional forged products. This combination is beneficial when fabricating complex products that require many forging steps. The WAAM technique was used by Shen et al. [25] to create iron-rich Fe-Al intermetallic alloys. They noted that the printed sample obtained a higher yield strength and a similar ductility compared to conventional powder metallurgy. Interestingly, the repeated heating step during the WAAM process created an in situ alloying phenomenon, leading to high alloy mixing. Additionally, Ti6Al4V produced using the plasma arc WAAM technique can outperform the aeronautical industry’s requirements, as shown by Veiga et al. [26]. Notably, the mechanical properties of the product present an anisotropy characteristic. Dong et al. [27] investigated Al-Zn-Mg-Cu alloy and revealed the existence of precipitation of nanoscale second phases during the WAAM process. In the vertical direction, the average tensile strength and yield strength are higher than in the horizontal direction [28]. The continuity and the porosity play as crack sources, creating the weakness of the alloy. According to Alonso et al. [29], Ti6Al4V alloy made by the WAAM technique requires more significant force, shorter chips, and shorter burr when compared to a traditional laminated plate. The reason is that the hardness of the WAAM sample is higher than the conventional laminated plate.

Besides controlling the electrical conditions, adjusting the printing trajectory can improve the 3D printing parts. For instance, Shembekar et al. [30] applied a 6-DOF robot to fabricate a 3D printing part with a collision-free trajectory. They achieved a good surface finish quality with reasonable printing time. Gardan et al. [31] increased the fracture toughness of the 3D printing sample by a principal stress direction design before printing. This special design improves fracture toughness by 20% compared to the classical method. Interestingly, Huang et al. [32] implied that special structures such as the Messerschmitt-Bölkow-Blohm and cantilever beams could enormously enhance the structural stiffness of the thermoplastic composite beam. Optimizing the trajectory during the multi-objects printing process can significantly decline the printing time, as presented in Luo et al. report [33]. Unlike traditional slicing software, this method calculated the optimal pathway between every two objects to create a continuous path, avoiding the collision phenomenon. The impacts of the printing angle between the feed orientation and the printing pathway tangent on the complex-shaped samples are discussed in the Kalashnikov et al. [34] report. The repeated heating cycle during the WAAM process leads to a coarsening in the grain structure, reducing the mechanical properties [35,36]. However, the effects of printing trajectory on the WAAM technique are rarely discussed. The investigation of printing trajectory is essential because various trajectories might result in different product characteristics.

In this report, we investigate the effects of printing trajectory on the microstructure and mechanical properties of the printed sample using the WAAM process. The WAAM process is carried out on a regular CNC machine and a MAG welding machine. The effects of the horizontal zigzag, lean zigzag, spiral, and curve zigzag strategy trajectories on the tensile properties and microstructure of the samples are investigated. The results could provide the optimal trajectory for the WAAM products.

## 2. Experimental Methods

**Figure 1:**
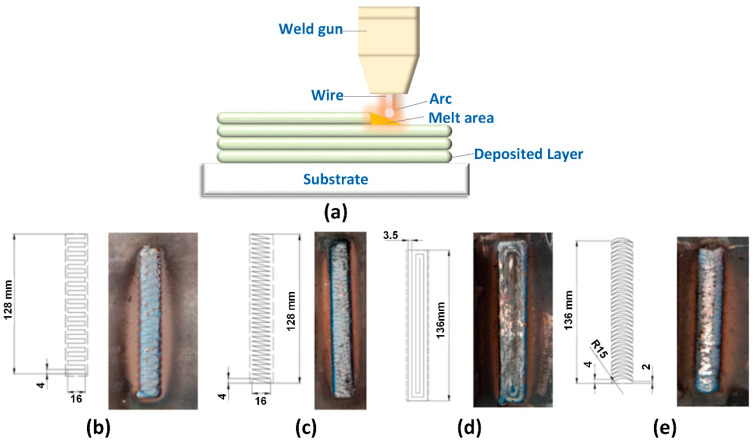
The WAAM process and the welding trajectories: (**a**) WAAM process, (**b**) strategy 1—horizontal zigzag, (**c**) strategy 2—lean zigzag, (**d**) strategy 3—spiral, and (**e**) strategy 4—curve zigzag.

Figure 1 shows the WAAM process and the welding strategy with different trajectories. The sample block is generated via printing layer by layer. In each layer, the weld gun moves in different trajectories, including strategy 1 with a horizontal zigzag trajectory, strategy 2 with a lean zigzag trajectory, strategy 3 with a spiral trajectory, and strategy 4 with a curve zigzag trajectory. The welding gun is located on a standard CNC machine and can move in different trajectories during printing. A steel base is prepared for printing the sample block. The steel base is cooled and fixed on the CNC machine to prevent the printed sample from being twisted due to the high thermal energy of the WAAM process. The mechanical and chemical properties of the S20C steel base are shown in Table 1 and Table 2. The welding machine uses steel wire with grade AWS A5.18 ER 70S-6, having a 0.8 mm diameter. The welding wire’s mechanical and chemical properties with grade AWS A5.18 ER 70S-6 are shown in Table 3 and Table 4. After machining sample blocks, they are machined to have three small samples in accordance with ASTM E8/E8M -13 standards before the tensile test. The steel blocks are cut to the tensile standard using wire electrical discharge machining (WEDM) to ensure dimension accuracy and avoid severe impact on the microstructure that could appear using traditional cutting methods. The welding parameters are selected as U = 22 V, I = 120 A, 400 mm/min travel rate, and 10 litter per min CO_2_ flow rate. In this study, the welding parameters are selected as U = 22 V, I = 120 A, 400 mm per min travel rate, and 10 litter per min CO_2_ flow rate. The weld heat input Q could be calculated as:

$$Q=\frac{U\times I}{Travel\;rate\;(\frac{mm}{s})}\times ƞ$$

where Q is the weld heat input (Joule/mm); ƞ is the weld thermal efficiency, which ranges from 69% to 91% for the GMAW technique. The calculated weld heat input Q in this study ranges from 274 J/mm to 360 J/mm, which is compatible with Kumar et al. [37,38] report. Moreover, the weld heat input depends on the sample thickness, wire diameter, and especially wire material. The target wall thickness also impacts the weld heat input value [39]. For the A5.18 ER 70S-6 welding wire, before experimenting, we also tried to test with some parameters that could create good samples. In this study, we focus on the welding strategy. In further investigation, we would try to survey more about the effect of the weld heat input for A5.18 ER 70S-6 welding wire.

The tensile test machine (SANS model CHT4106, China) is used to investigate the mechanical characteristics of the sample. In this study, the test pieces are horizontal to the steel base because the designed trajectory presents its impact most in this direction. The designed trajectory focuses on the horizontal factor. Therefore, we fabricated the size of the printing block suitable for the horizontal samples. Before observing the metallurgical microstructure, the samples are polished by the grinding polishing MP-2B machine, and etched by Nital 4% solution. The microstructure of these samples is tested using a scanning electron microscope (SEM5410 LV, JEOL, Japan) and a metallurgy microscope (Oxion OX.2153-PLM EUROMEX, Holland).

## 3. Results and Discussion

**Figure 2:**
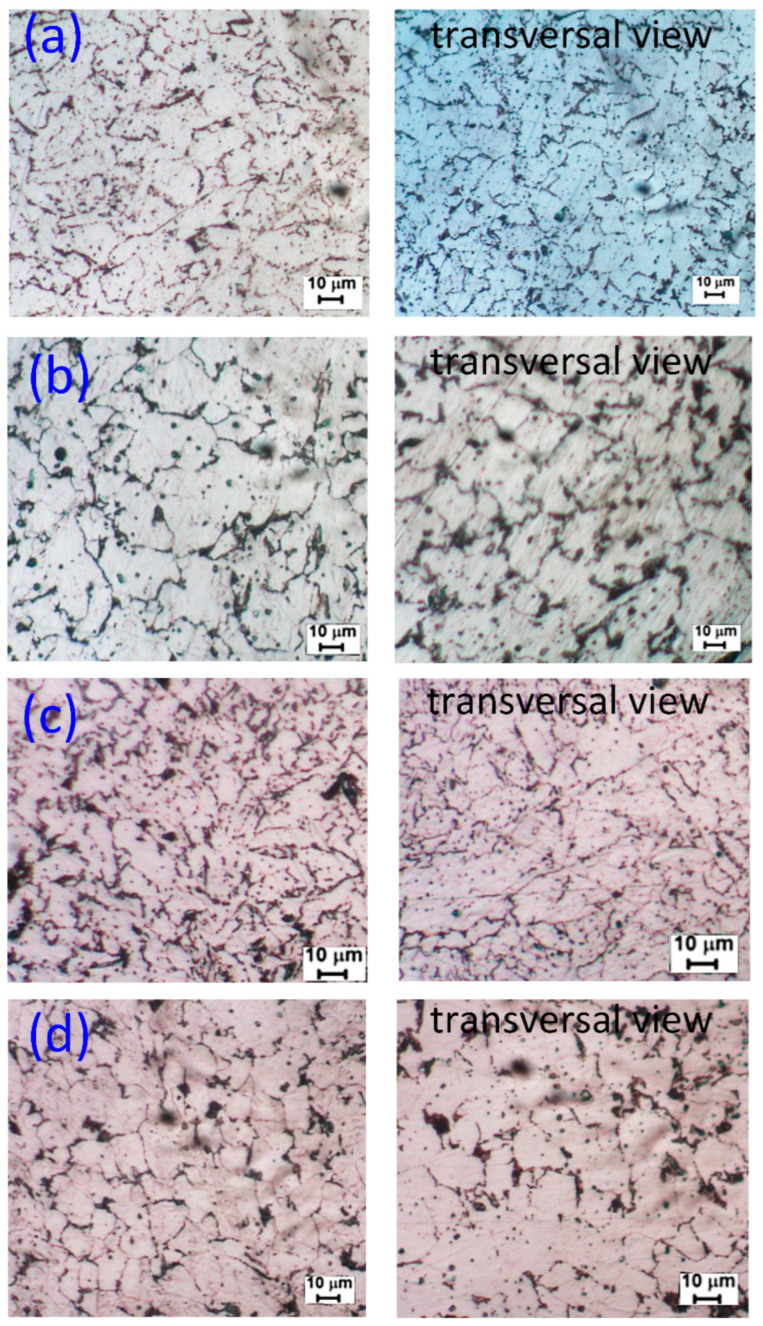
Microstructure of samples at different WAAM strategies: (**a**) strategy 1, (**b**) strategy 2, (**c**) strategy 3, and (**d**) strategy 4.

The metallurgy microstructures of the samples at different WAAM strategies are shown in Figure 2. The black phase that scatters on the matrix is pearlite, and the white matrix is ferrite. The dominant ferrite phase indicates that this sample is low-carbon steel, consistent with the wire grade AWS A5.18 ER 70S-6. Moreover, the microstructure images in a normal direction and their transverse images show that most grains on the microstructure have a polygonal and relatively equiaxial shape, consistent with other studies [40,41,42,43]. In Kumar et al. [38] report, they also generated a WAAM sample using AWS A5.18 ER 70S-6 welding wire and straight trajectory. The microstructure of those samples is also polygonal ferrite with a small amount of pearlite.

**Figure 3:**
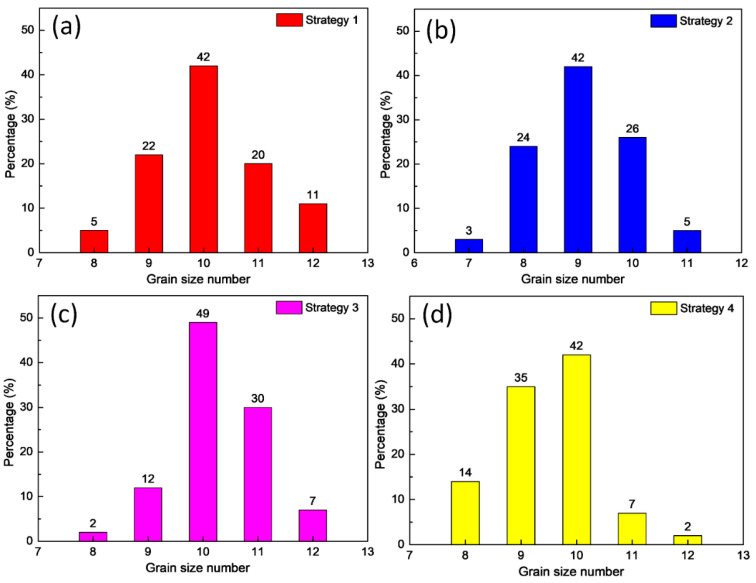
Grain size number distribution following ASTM E112-10 grain size number standard of samples at different WAAM strategies: (**a**) strategy 1, (**b**) strategy 2, (**c**) strategy 3, and (**d**) strategy 4.

Moreover, the microstructure test piece is machined from the printing block, not from connecting two steel parts. Therefore, we can conclude that the microstructure occurs at the center of the weld bead, presenting a relatively equiaxial shape. Notably, before being melted to create WAAM samples, the original welding wire has an anisotropy shape due to the initial drawing process of the steel wire. The melting of the solidification process enormously transforms the microstructure of the wire compared to the printing samples. From these figures, we can also analyze the grain size of these samples. The grain size number strongly affects the yield strength of the alloys [44]. To further investigate these microstructures, Figure 3 reveals the grain size number of the WAAM samples.

Figure 3 displays the grain size number distribution of WAAM samples at different WAAM strategies. The grain dimensions are measured via ImageJ software and classified using the ASTM E112-10 standard. Overall, the grain size numbers vary around 7–12. The average grain area values are 131 µm^2^, 245 µm^2^, 107 µm^2^, and 193 µm^2^, correspondings to samples of strategies 1, 2, 3, and 4. The results show that the WAAM strategy strongly impacts the grain size of the samples. Strategy 3 has the smallest grain size, while strategy 2 has the largest. The printing trajectory affects the level of cooling during the printing process, and different WAAM strategies produce different levels of cooling or solidification, affecting the sample grain size. Repeatedly and briefly heating the grain could make the grain size larger.

On the contrary, in the trajectory where the heating cycle is longer, the grain tends to become smaller. As seen in Figure 1d, strategy 3 with the spiral pathway makes it more time and space to release the thermal energy during the WAAM process. Therefore, it has the smallest grain size compared to other cases. Strategy 1 with horizontal zigzag also has a relatively small grain size compared to other cases. On the other hand, strategies 2 and 4 have much denser printing trajectories. Because the printing layers are strongly heated up during the printing process, the grain size has enough energy to grow, producing a coarser microstructure.

### Tensile Strength

**Figure 4:**
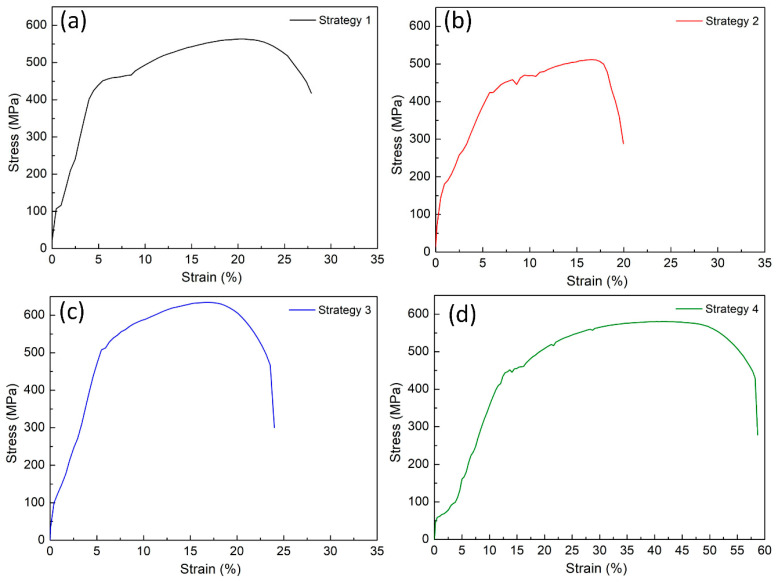
Stress-strain diagrams of samples at different WAAM strategies: (**a**) strategy 1, (**b**) strategy 2, (**c**) strategy 3, and (**d**) strategy 4.

**Figure 5:**
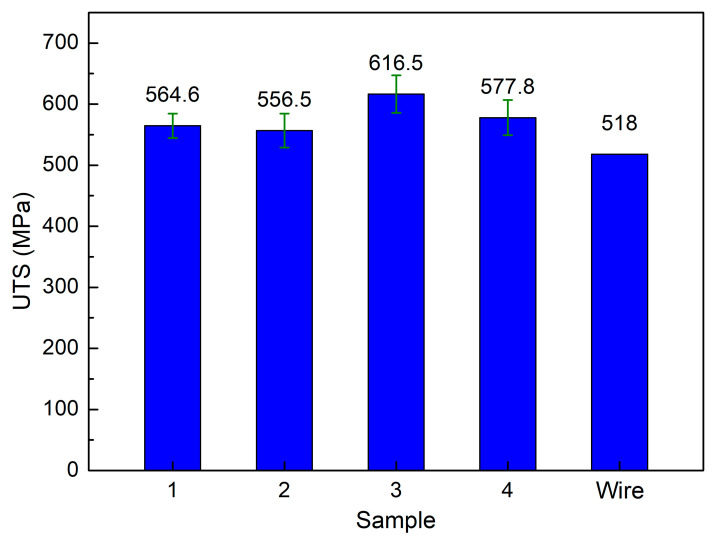
Average tensile strength of samples at different WAAM strategies.

Figure 4 demonstrates the tensile test diagram of samples at different WAAM strategies. The average values of the ultimate tensile strength (UTS) are summarized in Figure 5. The UTS values for strategies 1, 2, 3, and 4 are 564.6 MPa, 556.5 MPa, 616.5 MPa, and 577.8 MPa, respectively. Compared to the original wire’s UTS value of 496.4 MPa, the WAAM samples achieve an exceptionally higher UTS value, demonstrating the advantage of the WAAM technique. In Shirizly et al. [45] report, they also constructed a WAAM sample using AWS A5.18 ER 70S-6 welding wire and axisymmetric trajectory. The average tensile strength is 537 MPa, close to this study result and higher than the original wire. Compared to Gou et al. [46] study, which used the sample welding wire and straight trajectory, that research reached a UTS value of 500 MPa, which is also lower than this study’s result. The macro and microstructures produced from a designed trajectory combined with the melting and solidification process generate an advanced microstructure that enhances the UTS value. Strategy 1 and Strategy 4 have similar UTS values of 564.6 MPa and 577.8 MPa, respectively.

Furthermore, strategy 3 with spiral trajectory achieves the highest UTS value of 616.5 MPa, 19% higher than the original wire. This variation is due to the Hall-Petch equation, in which the smaller grain size presents a higher strength [44]. Because strategy 3 has the smallest grain size, it achieves the highest tensile strength value. The printing trajectory affects the level of cooling during the printing process [38]. Different WAAM strategies produce different cooling or solidification levels, affecting sample grain size. Repeatedly heating the grain for short periods could make the grain size larger.

On the contrary, in the trajectory where the heating cycle is longer, the grain tends to become smaller. As seen in Figure 1d, strategy 3 with the spiral pathway makes it more time and space to release the thermal energy during the WAAM process. Therefore, it has the smallest grain size compared to other cases. Strategy 2, with a lean zigzag trajectory, on the other hand, has the lowest UTS value, which is 7% lower than the original wire as it has the largest grain size, as presented in Figure 3. Besides, macrostructure and defects also impact sample characteristics.

**Figure 6:**
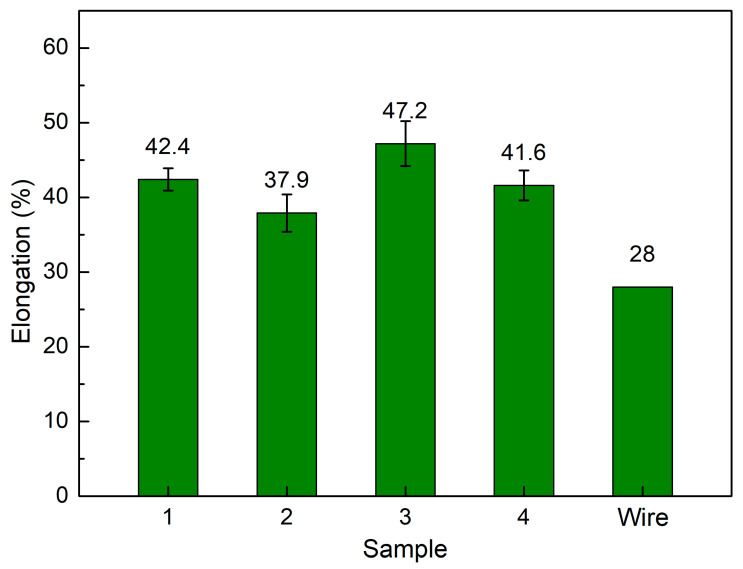
Average elongation of samples at different WAAM strategies.

Figure 6 shows the average elongation of samples at different WAAM strategies, WAAM samples have average elongation values of 42.4%, 37.9%, 47.2%, and 41.6%, corresponding to strategies 1, 2, 3, and 4, and these WAAM samples have significantly higher elongation values than the original wire, which is only 28%. With the same technique and axisymmetric trajectory, the WAAM sample could elongate by 24%, which is lower than this study’s results [37]. With a straight trajectory, the elongation of the WAAM sample could be 19%, which is also lower than this study, indicating the advance of complex trajectories in enhancing ductility. During the printing in each layer and between layers, the heating energy repeatedly anneals the sample; therefore, the sample is gradually softened, and the ductility is improved. The sample produced by strategy 3 has the highest elongation value. Strategy 2 has the lowest elongation value. Remarkably, the high UTS sample has a high or elongation value proportional to UTS. In other words, the sample has a higher UTS value and also a higher elongation value. The elongation values are also consistent with the grain size value, in which the smaller grain size has the higher elongation value, indicating the important role of grain size on the mechanical properties of the WAAM sample. The advancement of printing trajectory leads to an improvement in the WAAM sample elongation, similar to an increase in UTS value.

**Figure 7:**
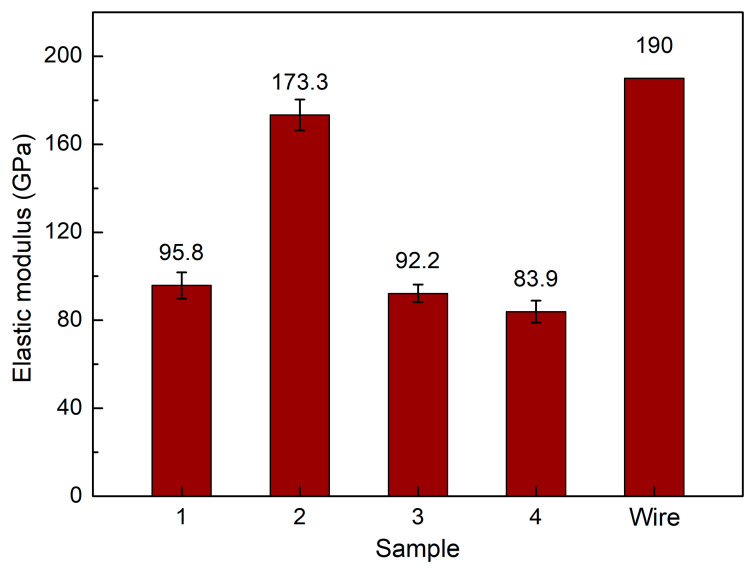
Average elastic modulus of samples at different WAAM strategies.

Figure 7 shows the average elastic modulus of samples at different WAAM strategies. WAAM samples have average elastic modulus values of 95.8 GPa, 173.3 GPa, 92.2 GPa, and 83.9 GPa, corresponding to strategies 1, 2, 3, and 4. Compared to the Gou et al. [46] study, which gains an elastic modulus of 208 GPa, this study has a lower elastic value. However, this study’s UTS and elongation values are higher than those that study. Only a sample with strategy 2 has a similar elastic modulus value to the original wire. This result is consistent with the elongation value, in which the lowest elongation sample has the highest elastic modulus of 173.3 GPa due to the stiffness of this sample. Besides, the trajectory of different strategies also impacts the elastic modulus value of the WAAM sample. Therefore, although the elongation values between these strategies are not so different, the elastic modulus of strategy 2 is significantly higher than in other cases. Other samples printed with strategies 1, 3, and 4 have substantially lower elastic modulus values than strategy 2. The strategy 4 sample has the lowest elastic modulus of 83.3 Gpa. The reason for this low elastic modulus value is related to the high elongation value compared to the original wire. As mentioned, the heating cycle of the WAAM process anneals the sample, resulting in a more ductile characteristic with higher elongation and lower elastic modulus.

**Figure 8:**
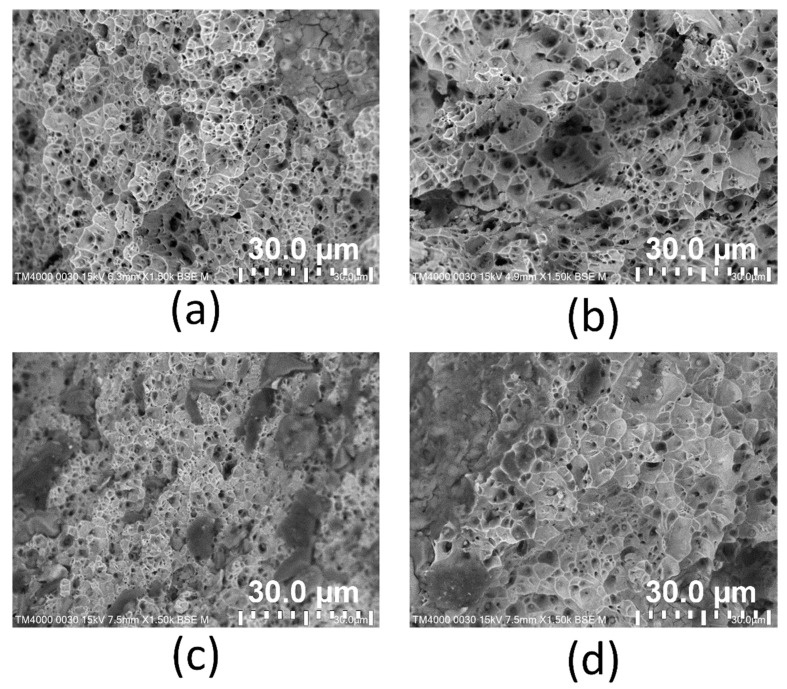
SEM fracture surface of samples at different WAAM strategies: (**a**) strategy 1, (**b**) strategy 2, (**c**) strategy 3, and (**d**) strategy 4.

After the tensile test, the fracture surface of the WAAM sample is observed via SEM microstructure. The SEM fracture surfaces of samples at different WAAM strategies are displayed in Figure 8. The failure in Figure 8 is observed in the fracture surface of the tensile test. In contrast, the tensile test piece is machined from the printing block, and therefore, the failure occurs at the center of the weld bead. The dimple shapes appear on the fracture surface of all samples, indicating the ductile characteristic of the WAAM samples [47]. These fracture surfaces’ equiaxial shape also represents the original microstructure’s equiaxial shape, as shown in Figure 2.

Additionally, the dimples’ size reveals the WAAM samples’ microstructure. Strategy 3 shows the smallest dimple size (Figure 8c), while strategy 2 presents the largest dimple size (Figure 8b). These results are consistent with the grain size, as shown in Figure 3.

## 4. Conclusions

This study surveys the effects of trajectory on the characteristics of WAAM steel samples. The following exciting notes to be mentioned are:

- The grains in the WAAM samples are isotropic, and their grain size number values range from about 7 to 12. The grain size of strategy 3 with spiral trajectory is the smallest, while strategy 2 with lean zigzag has the largest. This difference could result from the printing process’s heat input or storage;
- The WAAM samples achieve a significantly higher UTS value than the original wire, proving the benefit of the WAAM technique. The highest UTS value, 616.5 MPa, is attained by strategy 3 with a spiral trajectory. The lowest UTS value for strategy 2, which has a lean zigzag trajectory, is 556.5 Mpa;
- WAAM samples have significantly higher elongation values than the original wire, with an elongation value of only 22%. Strategy 3 produced the sample with the highest elongation value of 47.2%, and the elongation value of Strategy 2 was 37.9%. The elongation value is proportional to the UTS value;
- WAAM samples have average elastic modulus values of 95.8 GPa, 173.3 GPa, 92.2 GPa, and 83.9 GPa, corresponding to strategies 1, 2, 3, and 4. Only a sample with strategy 2 has a similar elastic modulus value to the original wire;
- The dimples shape appears on the fracture surface of all samples, indicating that the WAAM samples are ductile. The equiaxial shape of these fracture surfaces corresponds to the original microstructure’s equiaxial shape. The results provide the optimal trajectory for the WAAM products is a spiral trajectory, while the lean zigzag trajectory only gains modest mechanical characteristics.

## Figures and Tables

**Table 1:** Mechanical properties of S20C (JIS) steel base.

Tensile strength	min 423 MPa
Yield strength	min 311 MPa
Elongation	min 35.4%
Elastic modulus	190 GPa

**Table 2:** Chemical composition of S20C steel base.

Weight %	C	Si	Mn	P	S	Ni	Cr	Cu
ER 70S-6	0.18–0.23	0.15–0.35	0.3–0.6	0.03 max	0.035 max	0.2 max	0.2 max	0.3 max

**Table 3:** Mechanical properties of welding wire grade AWS A5.18 ER 70S-6.

Tensile strength	518 MPa
Yield strength	424 MPa
Elongation	28%
Elastic modulus	190 GPa

**Table 4:** Chemical composition of welding wire grade AWS A5.18 ER 70S-6.

Weight %	C	Mn	Si	P	S	Ni	Cr	Mo	V	Cu
ER 70S-6	0.06–0.15	1.40–1.85	0.80–1.15	0.025 max	0.035 max	0.15 max	0.15 max	0.15 max	0.03 max	0.05 max

## Data Availability

The data used to support the findings of this study are available from the corresponding author upon request.
